# In vivo brain estrogen receptor density by neuroendocrine aging and relationships with cognition and symptomatology

**DOI:** 10.1038/s41598-024-62820-7

**Published:** 2024-06-20

**Authors:** Lisa Mosconi, Matilde Nerattini, Dawn C. Matthews, Steven Jett, Caroline Andy, Schantel Williams, Camila Boneu Yepez, Camila Zarate, Caroline Carlton, Francesca Fauci, Trisha Ajila, Silky Pahlajani, Randolph Andrews, Alberto Pupi, Douglas Ballon, James Kelly, Joseph R. Osborne, Sadek Nehmeh, Matthew Fink, Valentina Berti, Jonathan P. Dyke, Roberta Diaz Brinton

**Affiliations:** 1 Department of Neurology, Weill Cornell Medicine, 402 East 70th Street, LH-404, New York, NY 10021 USA; 2 Department of Radiology, Weill Cornell Medicine, New York, NY USA; 3 Nuclear Medicine Unit, Department of Biomedical Experimental and Clinical Sciences “Mario Serio”, University of Florence, Florence, Italy; 4 ADM Diagnostics, Grayslake, IL USA; 5 Department of Population Health Sciences, Weill Cornell Medicine, New York, NY USA; 6 Department of Pharmacology and Neurology, University of Arizona, Tucson, AZ USA

**Keywords:** Endocrinology, Neurology, Neuroscience, Cognitive ageing, Neural ageing

## Abstract

17β-estradiol, the most biologically active estrogen, exerts wide-ranging effects in brain through its action on estrogen receptors (ERs), influencing higher-order cognitive function and neurobiological aging. However, our knowledge of ER expression and regulation by neuroendocrine aging in the living human brain is limited. This in vivo brain ^18^F-fluoroestradiol (^18^F-FES) Positron Emission Tomography (PET) study of healthy midlife women reveals progressively higher ER density over the menopause transition in estrogen-regulated networks. Effects were independent of age, plasma estradiol and sex hormone binding globulin, and were highly consistent, correctly classifying all women as being postmenopausal or premenopausal. Higher ER density in target regions was associated with poorer memory performance for both postmenopausal and perimenopausal groups, and predicted presence of self-reported mood and cognitive symptoms after menopause. These findings provide novel insights on brain ER density modulation by female neuroendocrine aging, with clinical implications for women’s health.

## Introduction

Estrogen is a class of steroid hormones that play a key role in the development and regulation of the female reproductive system. Additionally, burgeoning evidence documents that 17β-estradiol (E2), the most biologically active form of estrogen, exerts wide-ranging effects on neurological and cognitive functions^1,2^, as well as neurodevelopmental and neurodegenerative processes^2,3^.

E2 signals through estrogen receptors (ERs) that are ubiquitously distributed in brain^1,2^ where they coordinate neuroprotective signaling cascades^4^ and modulate cerebral blood flow, energy metabolism, inflammation, and oxidative processes^5,6^. This is fundamental for humans, as all women experience a drop in circulating E2 as they undergo menopause, either through the endocrine aging process or through medical intervention^7^. Given the integrative role of E2 for brain function^1,2^, it is not surprising that many symptoms of menopause are neurological in origin, manifesting as changes in thermoregulation, mood, sleep and cognition^3,8^.

Hypoestrogenic postmenopausal women are also more vulnerable to brain injury, affective disorders, and certain neurodegenerative conditions such as Alzheimer's disease (AD) relative to premenopausal women and to men of similar age^9^. These conditions are influenced by ER dysregulation^4^. In translational brain imaging studies, the menopause transition is associated with reduced gray matter volume (GMV) and glucose metabolism^10–14^, as well as amyloid-beta (Aβ) plaques and tau pathology^10–12,15,16^. Postmenopausal GMV and metabolic declines are attenuated by estrogen therapy^17,18^, suggesting that ER-mediated neurological processes retain dynamic properties well into menopause.

Despite preclinical evidence of the importance of ERs for neural function and the increasing use of estrogen therapies in clinical practice, our direct knowledge of ER activity in the human brain is very limited.

Positron emission tomography (PET) is the only technique currently available that enables in vivo assessment of ER expression. 16α-^18^F-fluoro-17β-estradiol (^18^F-FES) is the most utilized ER ligand in oncology, exhibiting selective binding affinity for ERs, especially ER alpha (ERα)^19^, and high signal correlation with ER expression in tumors^20,21^. However, ^18^F-FES studies of brain ER expression are scarce, with most conducted in non-human animals. In female rodents, ^18^F-FES uptake was evident in brain ER-rich regions, chiefly pituitary, hypothalamus, striatum, limbic lobe, and cortex^22–24^, and increased following bilateral oophorectomy^22,23^. To our knowledge, the only brain ^18^F-FES PET study in neurologically intact humans was conducted in breast cancer patients, showing trends toward higher pituitary uptake in postmenopausal as compared to premenopausal patients^23^. However, results were not corrected by age^23^. A few case reports also indicate specific ^18^F-FES signal in pituitary and hypothalamus of breast cancer patients and postmenopausal controls^25,26^.

Herein, we took a translational approach to systematically investigate brain ER density and its modulation by neuroendocrine aging using ^18^F-FES PET in healthy midlife women at different menopausal stages. Additionally, we examined the relationships of ER density with cognition and presence of menopausal symptoms of neurological origin.

## Results

To characterize ER distribution and its modulation by neuroendocrine aging, we performed ^18^F-FES PET imaging on 54 consecutive midlife women, aged 40–65 years, divided into three groups of 18 participants each balanced by menopausal stage: premenopause (scanned at midcycle), perimenopause, postmenopause. As described in the Methods, we used graphic Logan plots^27^ to derive ^18^F-FES distribution volume ratios (DVR) relative to cerebellar gray matter, reflecting ER density in a priori selected ER-rich regions-of-interest (ROI) including pituitary, hypothalamus, amygdala, hippocampus, caudate nucleus, thalamus, posterior cingulate cortex (PCC), and frontal cortex^28–31^.

There were no differences in demographic, clinical and cognitive test measures between groups, except for an age difference between the premenopausal and postmenopausal groups (Table 1). This difference was addressed according to published protocols^12,32^ (Methods), showing age-independent effects of menopause status on ER density, which were not modified by age (see Sensitivity analysis). All analyses were adjusted for age, plasma E2 and sex-hormone binding globulin (SHBG) levels.

**Table 1:** Participant characteristics.

	Premenopause	Perimenopause	Postmenopause
N	18	18	18
Age, years	45(4)	50(4)	55(4)*
Education, years	18(2)	17(2)	17(2)
Race, % white	67	90	89
MoCA score, unitless	28(2)	28(2)	28(2)
Hypertension, % positive	0	11	6
Diabetes, % positive	0	0	6
History of mild depression, % positive	22	11	11
Smoking, % never smoker	83	72	78
Plasma estradiol, E2 (pg/mL)	159(29)	45(20)*	18(26)*
Plasma sex hormone binding globulin, SHBG (nmol/L)	118(12)	85(8)	57(11)*
Cognitive scores, unitless:			
Logical memory, immediate recall	15.1(1.3)	15.1(0.9)	14.8(1.2)
Logical memory, delayed recall	13.6(1.5)	13.2(1.0)	13.1(1.4)
Animal naming	25.3(1.7)	24.5(1.2)	23.7(1.7)
Object naming	13.8(0.4)	14.6(0.3)	14.2(0.4)
FAS	50.8(4.1)	52.6(2.8)	46.0(4.0)
RAVLT total	30.2(1.9)	29.0(1.3)	28.6(1.9)
RAVLT delayed recall	11.1(1.0)	9.50(0.7)	10.0(0.9)
RAVLT recognition	14.1(0.5)	13.9(0.3)	13.8(0.5)
Trail Making Test B	52.8(6.0)	53.8(4.1)	63.0(5.9)

Values are means (standard deviation) or percentages (%) as indicated. Plasma hormone measures are age-adjusted means (standard error). Cognitive measures are age and education-adjusted means (standard error).

*MoCA* montreal cognitive assessment, *RAVLT* rey auditory verbal learning test.

*Different from the premenopausal group at *P* < 0.05.

### ^18^F-FES time activity curves and distribution patterns in brain

**Figure 1:**
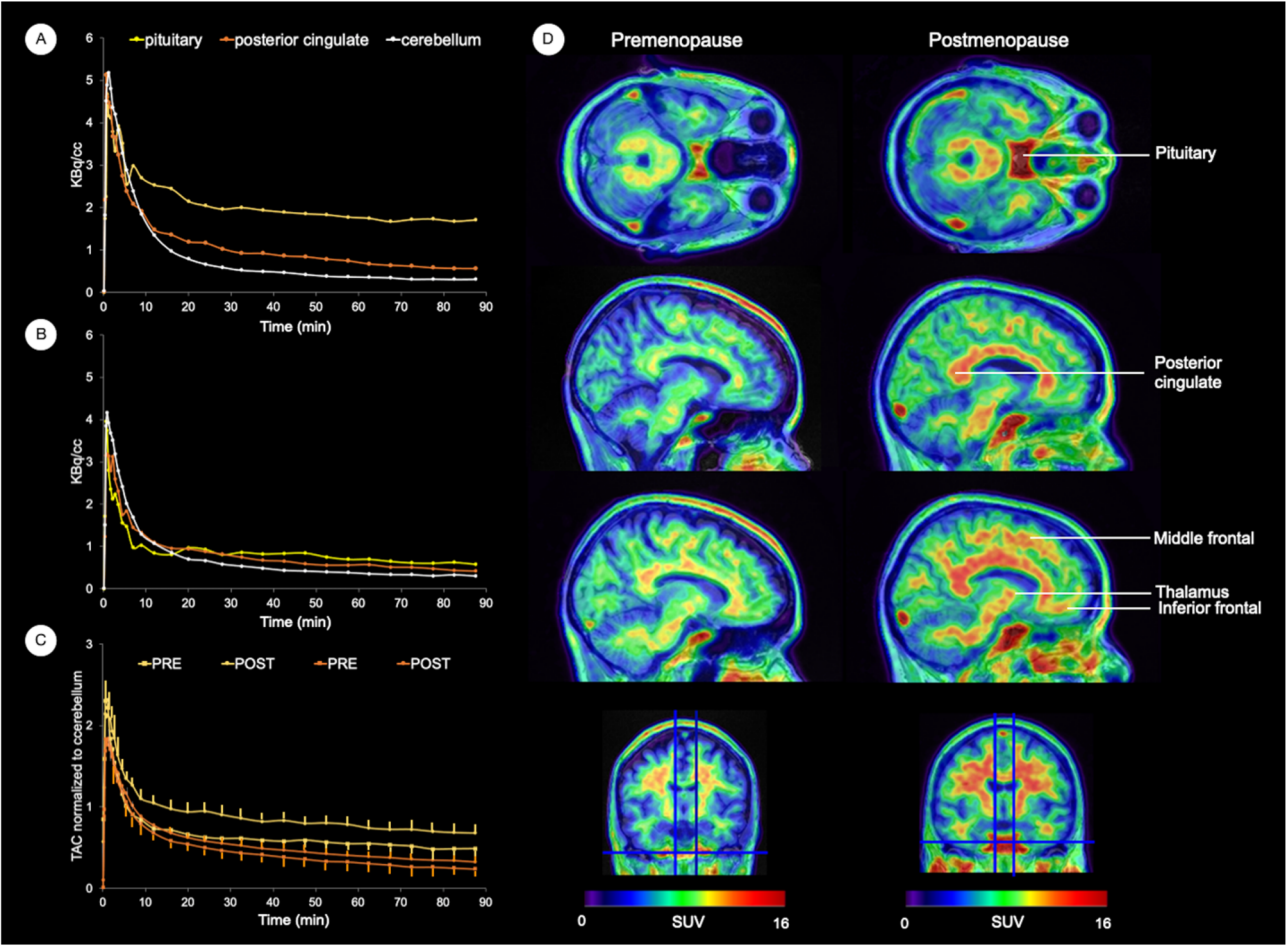
Brain [18F]Fluoroestradiol time activity curves and distribution. Left panel: Time-activity curves (TACs) of ^18^F-Fluoroestradiol (^18^F-FES) in target estrogen receptor (ER)-rich brain regions. Representative TACs are shown in pituitary, posterior cingulate and cerebellum from representative (**A**) postmenopausal and (**B**) premenopausal participants. (**C**) Mean TACs (standard errors) in pituitary and posterior cingulate normalized by cerebellar measures are displayed for the entire postmenopausal (squares) and the premenopausal (circles) groups. Right panel: ^18^F-FES PET images (summed frames over 30–90 min, pseudo-colored using a rainbow spectrum scale) overlaid on the coregistered structural MRI of the two representative premenopausal and postmenopausal participants in (**A**) and (**B**). PET images are scaled identically, with a range of 0–16 standardized uptake values (SUV) for each participant (see color-coded scales), depicting higher tracer uptake in ER-rich regions in the postmenopausal participant. From top to bottom, ^18^F-FES PET images are displayed in the axial, sagittal and coronal views at the level of pituitary, posterior cingulate, frontal cortex and thalamus. Comparatively low to negligible uptake is evident in the lateral inferior cerebellar gray matter, which was used as the reference region for quantification. Note that tracer uptake is also present in portions of the cortical and cerebellar white matter, as well as corpus callosum and brainstem, which was generally higher in postmenopausal than in premenopausal women across the entire dataset. However, since ^18^F-FES white matter uptake is predominantly non-specific^25,26^, white matter regions were excluded from our statistical analysis.

On examination of time activity curves (TACs), the ^18^F-FES tracer showed fast brain penetration reaching peak values within less than 2 min, followed by a relatively rapid clearance stage followed by retention in target regions, where activity curves (decay corrected) showed steady-state kinetics by approximately 30 min post-injection (Fig. 1A–C). Tracer distribution in the late time-frames was somewhat heterogeneous, with the highest levels of radioactivity accumulation seen in pituitary, and moderate levels in PCC, thalamus, and caudate. Medial temporal and frontal levels were comparatively lower, but distinct from the cerebellar reference.

On visual examination of ^18^F-FES PET scans, the regional distribution pattern illustrated in Fig. 1D was observed in virtually all of the participants, with uptake differences evident between postmenopausal and premenopausal statuses. The pituitary exhibited the strongest signal, followed by PCC, thalamus, basal ganglia and frontal cortex. Comparatively low or negligible uptake in lateral inferior cerebellar gray matter was evident on all scans, confirming this subregion’s suitability as a reference for graphic Logan analysis^27^. Tracer uptake was also present in portions of the cortical and cerebellar white matter, as well as corpus callosum and brainstem. Qualitatively, tracer uptake in white matter was generally higher in the postmenopausal group than in the premenopausal group, as exemplified in Fig. 1D. However, based on previous findings that ^18^F-FES uptake in white matter is predominantly non-specific^25,26^, white matter regions were excluded from our statistical analysis.

### ER density by menopause status

**Figure 2:**
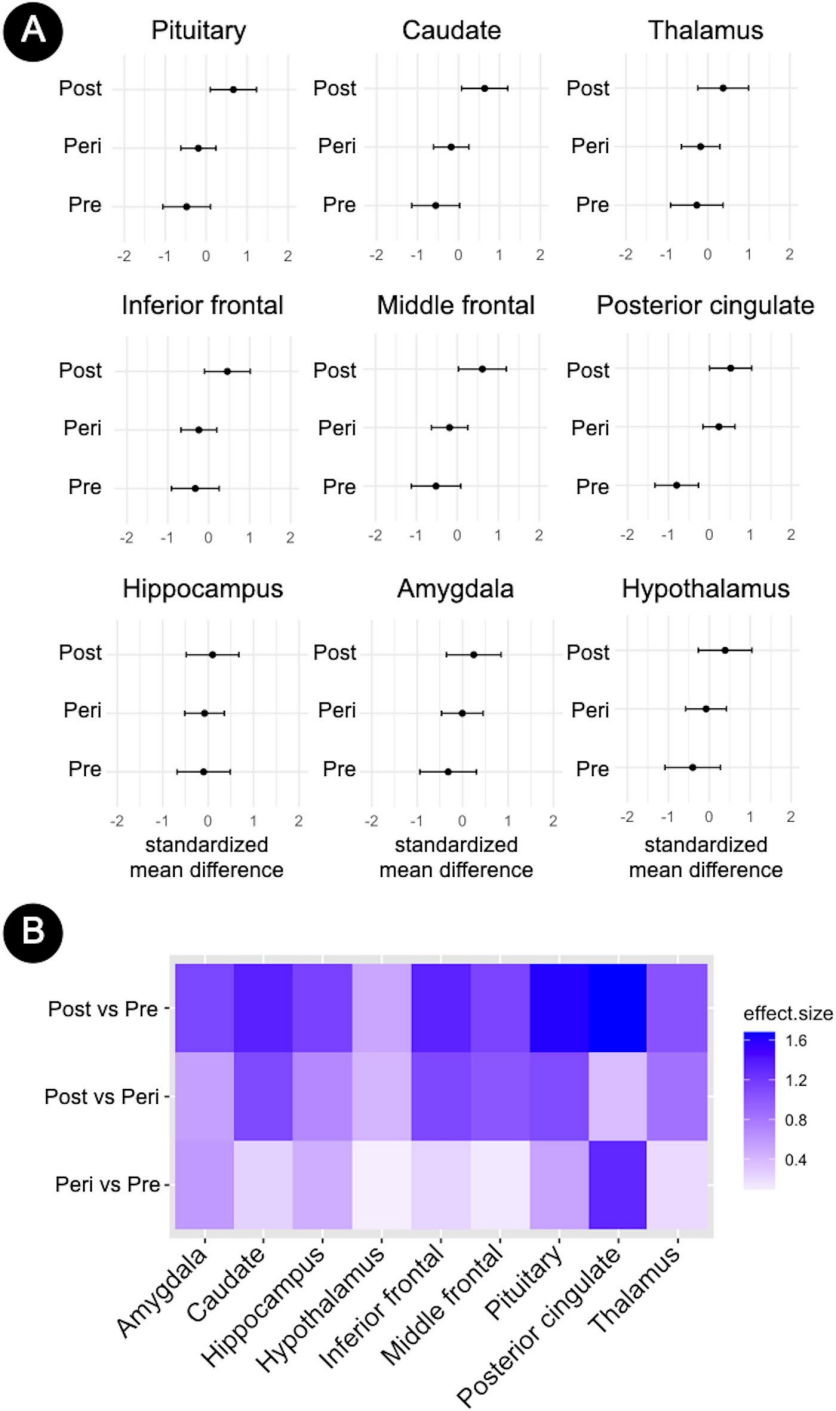
Regional brain estrogen receptor density by menopause stage. (**A**) Forest plots showing standardized brain ^18^F-FES distribution volume ratios (DVR) in pre-specified regions of interest by menopausal status, adjusted by age, plasma estradiol (E2) and sex hormone binding globulin (SHBG). Error bars are 95% confidence intervals. (**B**) Heatmaps showing standardized pairwise mean regional ^18^F-FES DVR differences between menopause statuses expressed as Cohen’s d coefficients, where d ≥ 0.8 reflects a large effect size. Abbreviations: Peri, perimenopause; Post, postmenopause; Pre, premenopause.

^18^F-FES DVRs generally increased in a menopause-stage dependent fashion, reflecting higher ER density (Fig. 2a). Adjusting for age, plasma E2 and SHBG, regional measures were highest in the postmenopausal group, intermediate in the perimenopausal group, and lowest in the premenopausal group (Table 2, Fig. 2a).

**Table 2:** Regional brain estrogen receptor density by menopausal stage.

Region	Premenopausal group	Perimenopausal group	Postmenopausal group
Amygdala	1.072 (0.019)	1.091 (0.014)	1.107 (0.018)
Caudate	1.305 (0.023)	1.334 (0.017)	1.398 (0.022)*
Hippocampus	1.132 (0.017)	1.133 (0.013)	1.144 (0.017)
Hypothalamus	0.955 (0.031)	0.984 (0.023)	1.027 (0.030)
Inferior frontal gyrus	1.165 (0.017)	1.170 (0.013)	1.211 (0.016)
Middle frontal gyrus	1.142 (0.020)	1.164 (0.015)	1.218 (0.020)*
Pituitary	2.047 (0.185)	2.230 (0.136)	2.777 (0.179)*^
Posterior cingulate cortex	1.375 (0.023)	1.466 (0.017)*	1.498 (0.023)*
Thalamus	1.401 (0.024)	1.408 (0.018)	1.449 (0.023

Means (standard error) by menopause status, adjusted by age, plasma estradiol (E2) and sex hormone binding globulin (SHBG) levels. ^18^F-FES data are distribution volume ratios (DVR) to cerebellar gray matter.

*Different from premenopausal group; ^different from perimenopausal group, at *P* < 0.05.

On multivariate analysis, the postmenopausal group exhibited overall higher DVR across brain regions than the premenopausal group (multivariable adjusted *P* = 0.009), and marginally higher DVR than the perimenopausal group (multivariable adjusted *P* = 0.086) (Table 2, Fig. 2a). On a regional basis, the postmenopausal group exhibited higher DVR in pituitary as compared to both premenopausal and perimenopausal groups, as well as higher DVR in PCC and caudate as compared to the premenopausal group (multivariable adjusted *P* ≤ 0.05) (Table 2, Fig. 2a).

There were no overall differences between perimenopausal and premenopausal groups (multivariable adjusted *P* = 0.328). On univariate analysis, the perimenopausal group exhibited higher DVR in PCC as compared to the premenopausal group (multivariable adjusted *P* = 0.009) (Table 2, Fig. 2a).

### Prediction of group membership

Pituitary, caudate, PCC and middle frontal DVR yielded the largest effect sizes in differentiating postmenopausal from premenopausal participants (Cohen’s d’s ≥ 1.28, Fig. 2b). DVR in these regions resulted in 100% predictive accuracy in classifying participants as being postmenopausal or premenopausal.

### Associations of ER density with cognition

In the entire sample, higher DVR in regions with established cognitive functions, e.g. hippocampus, amygdala, PCC and frontal cortex, were associated with lower scores on logical memory delayed recall (multivariable adjusted *P* ≤ 0.038; Table 3). DVR in hippocampus and amygdala also correlated with lower logical memory immediate recall (multivariable adjusted *P* ≤ 0.023; Table 3).

**Table 3:** Associations of brain estrogen receptor density with cognitive performance.

	Logical memory, immediate	*P*	Logical memory, delayed	*P*	Trail Making Test B	*P*	Animal naming	*P*	Object naming	*P*
Overall										
Amygdala	− 0.311	**0.023**	− 0.411	**0.002**	0.092	0.514	0.168	0.230	− 0.041	0.768
Hippocampus	− 0.468	** < 0.001**	− 0.540	** < 0.001**	0.037	0.795	− 0.064	0.651	− 0.085	0.544
Inferior frontal	− 0.149	0.278	− 0.262	***0.058***	0.110	0.433	0.124	0.556	− 0.057	0.683
Middle frontal	− 0.195	0.162	− 0.286	**0.038**	0.128	0.359	− 0.083	0.553	0.011	0.939
Posterior cingulate	− 0.194	0.163	− 0.296	**0.032**	0.180	0.556	− 0.083	0.378	− 0.130	0.352
Perimenopause										
Amygdala	− 0.314	0.220	− 0.373	0.141	− 0.139	0.596	− 0.182	0.484	0.303	0.238
Hippocampus	− 0.594	**0.012**	− 0.498	**0.042**	− 0.047	0.857	0.003	0.99	0.331	0.195
Inferior frontal	0.135	0.606	− 0.067	0.798	− 0.338	0.185	0.125	0.632	0.423	0.091
Middle frontal	− 0.154	0.554	− 0.155	0.553	− 0.024	0.927	− 0.262	0.31	0.216	0.404
Posterior cingulate	0.067	0.799	0.058	0.826	− 0.196	0.451	0.001	0.998	− 0.266	0.302
Postmenopause										
Amygdala	− 0.104	0.682	− 0.384	0.115	0.387	0.112	0.163	0.518	− 0.264	0.290
Hippocampus	− 0.210	0.403	− 0.475	**0.046**	0.332	0.178	− 0.282	0.256	− 0.139	0.582
Inferior frontal	− 0.175	0.488	− 0.471	**0.049**	0.308	0.214	0.199	0.427	− 0.164	0.515
Middle frontal	− 0.185	0.463	− 0.478	**0.045**	0.266	0.286	0.303	0.221	− 0.212	0.399
Posterior cingulate	− 0.278	0.264	− 0.564	**0.015**	0.424	***0.080***	− 0.049	0.846	− 0.336	0.172

Partial correlation coefficients and associated *P* values from linear regression models. Significant *P* values are in bold, trends are in italics.

In stratified analysis, the postmenopausal group exhibited negative associations between hippocampus, PCC and frontal regions DVR and logical memory delayed recall (multivariable adjusted *P* ≤ 0.049), and borderline associations between PCC DVR and TMT-B scores (multivariable adjusted *P* = 0.080) (Table 3). The perimenopausal group exhibited negative associations between hippocampal DVR and logical memory immediate and delayed recall (multivariable adjusted *P* ≤ 0.042; Table 3).

### Associations of ER density with menopause symptoms

**Figure 3:**
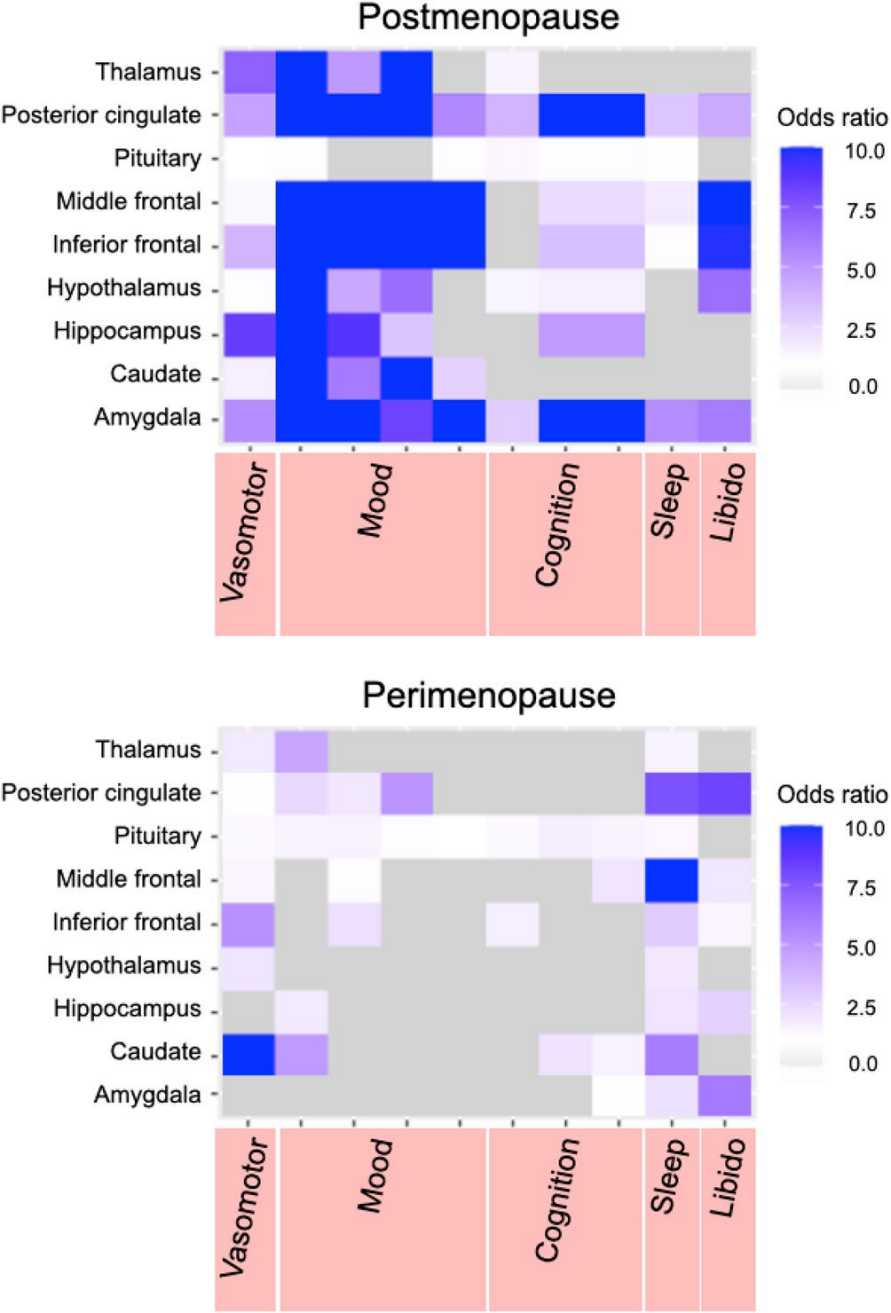
Associations between brain estrogen receptor density and menopause symptoms. Heatmaps show associations between ^18^F-FES distribution volume ratios (DVR), reflecting estrogen receptor (ER) density, and presence of self-reported menopause symptoms by domain. From left to right, vasomotor symptoms (hot flashes, night sweats), mood symptoms (low mood, mood fluctuations, tearfulness, irritability), cognition (reduced focus, memory complaints, brain fog), disturbed sleep, and low libido. Multivariable-adjusted odds ratios (OR) are displayed on a purple-to-grey color-coded scale, with purple indicating positive associations and grey indicating neutral associations. Estimates are presented separately for the postmenopausal and perimenopausal groups.

ER density exhibited generally positive associations with self-reported presence of menopausal symptoms at the postmenopausal stage, which reached significance for disturbed mood and subjective cognitive declines (Fig. 3). In the postmenopausal group, regional DVR were consistently associated with current presence of mood symptoms, with significant effects in amygdala [OR 10, 95% C.I. 2.677, > 10], frontal regions [OR 10, 95% C.I. 2.139, > 10], PCC [OR 10, 95% C.I. 4.031, > 10] and thalamus [OR 10, 95% C.I. 2.434, > 10]. PCC DVR also predicted presence of memory complaints [OR 10, 95% C.I. 1.122, 10] (Fig. 3). In the perimenopausal group, associations between DVRs and presence of menopausal symptoms did not reach significance (Fig. 3).

### Sensitivity analysis

#### Voxel-based analysis

**Figure 4:**
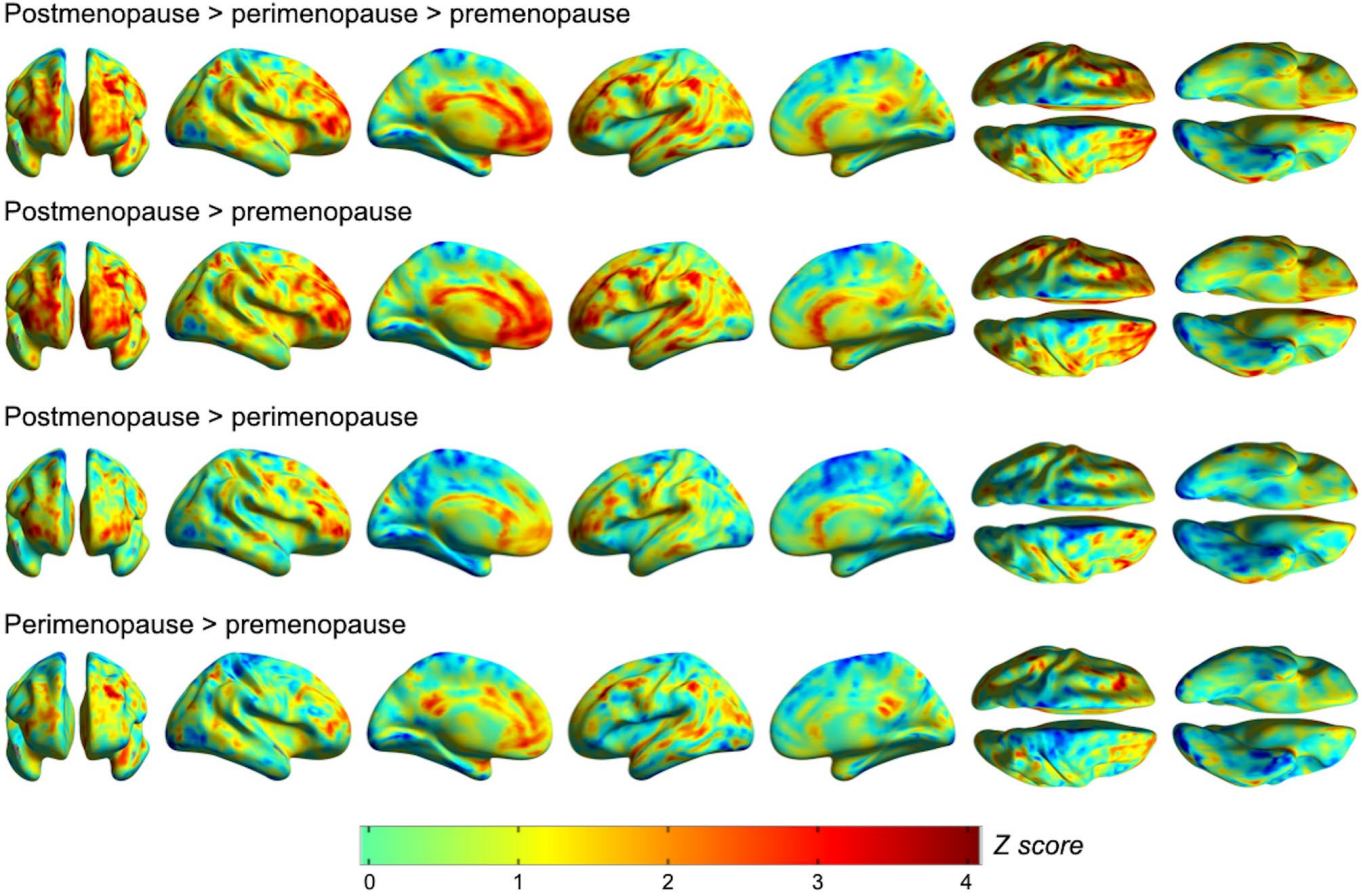
Voxel-wise analysis of brain estrogen receptor density by menopause stage. Surface statistical parametric maps (SPMs) of voxel-wise differences in parametric ^18^F-FES binding potential images, reflecting estrogen receptor (ER) density, between premenopausal, perimenopausal and postmenopausal groups, at *P* < 0.05 cluster-level corrected for family-type wise error (FWE), adjusted by age, plasma estradiol (E2) and sex hormone binding globulin (SHBG). SPMs are represented on a spectrum color-coded scale with corresponding Z scores. Corresponding statistics are reported in Supplementary Table 1.

Statistical Parametric Mapping (SPM12)^33^ was used to carry out exploratory analyses of parametric ^18^F-FES images in frontal, temporal, parietal and cingulate cortices on a voxel-by-voxel basis, adjusting by confounders. Results confirmed presence of progressively higher DVR from premenopausal to postmenopausal stages, with some effects of hemispheric laterality (*P* < 0.05, cluster-level corrected for family-type wise error, FWE; Fig. 4). Menopause status was associated with DVR in middle frontal gyrus, bilaterally, as well as in caudate and anterior cingulate of the left hemisphere, and superior frontal gyrus of the right hemisphere (*P*_*FWE*_ ≤ 0.043, Fig. 4 and Supplementary Table 1).

On post-hoc analysis, the postmenopausal group exhibited higher DVR in these regions as compared to both premenopausal and perimenopausal groups (*P*_*FWE*_ ≤ 0.043, Fig. 4 and Supplementary Table 1). The perimenopausal group exhibited higher DVR in cingulate cortex and inferior parietal lobule of the left hemisphere as compared to the premenopausal group (*P*_*FWE*_ ≤ 0.006, Fig. 4 and Supplementary Table 1).

For completeness, we also examined the results at *P* < 0.001, uncorrected. This identified additional clusters with higher DVR in insula, precentral gyrus, anterior cingulate, inferior parietal lobule, superior and inferior frontal gyri of the left hemisphere in the postmenopausal group compared to the premenopausal group (Supplemental Table 2). Progressively higher DVR from premenopausal to postmenopausal stages were also observed in bilateral superior frontal gyri, left insula, and left caudate (Supplemental Table 2).

#### Age-based modification effects

Across the entire sample, age was positively associated with hippocampal, amygdala, PCC and thalamus DVR (*P* ≤ 0.035), and exhibited no significant associations in the other regions (*P* > 0.108; Supplementary Table 3). On examination of menopausal status, there were no significant associations between age and DVR in any region of any group (Supplementary Table 3). There were no significant age-based modification effects on the associations of menopause status and ER density (Supplementary Table 4).

## Discussion

This in vivo multi-modality imaging study demonstrates progressively higher brain ^18^F-FES DVR, reflecting higher ER density, over the menopause transition, independent of chronological age, plasma E2 and SHBG. Results demonstrate high anatomical overlap with estrogen-regulated brain networks involved in both reproductive and higher-order cognitive functions. Elevations in ER density were consistent, correctly classifying all women as being postmenopausal or premenopausal. ER density in regions subserving cognitive functions, chiefly hippocampus, was associated with lower memory scores for both postmenopausal and perimenopausal groups. Additionally, ER density predicted presence of self-reported mood and cognitive symptoms among postmenopausal women.

For decades, the classic view of estrogen action in brain was confined to regulation of ovulation and female reproductive behavior^1,2^. Later studies challenged this paradigm, identifying E2 as the “master regulator” of neurological function in female brain due to its broad impact on multiple neural processes^2,3^. Further advances led to discovery that E2 actions are mediated by specialized brain ERs, including classical ERα and ERβ, which are found in neurons, glial cells, and astrocytes; and G protein–coupled estrogen receptor 1 (GPER1) mainly located in plasma membranes^1,2^. ERs modulate synaptic plasticity, adult neurogenesis, and DNA repair^4^, as well as expression of a wide variety of genes linked to lipid metabolism, vasodilatation, synaptic potentiation, and myelination^1,2^. Major projection neurons such as cholinergic, serotonergic, and dopaminergic systems are also responsive to ER activity^5,6^, further highlighting the importance of ER-mediated activity for brain health.

However, our knowledge of ER expression in the living human brain is very limited. PET imaging with ER ligands such as ^18^F-FES PET is the only technique currently available to assess ER expression in vivo. Brain ^18^F-FES PET studies in rodents have been instrumental in demonstrating specific tracer binding in regions with known ERα expression, chiefly pituitary and hypothalamus^22,23,34^, and lower yet measurable signal in striatum, limbic lobe, and cortex^24^. In female rats, E2 declines following bilateral oophorectomy provoked a marked increase in ER density in pituitary and hypothalamus relative to non-oophorectomized controls^22,23^, as well as smaller increases in amygdala and frontal cortex^22^. Regional ER upregulation was reduced by pre-surgical administration of E2^22^.

Whether similar mechanisms are active in women’s brains is unknown. Currently, the only brain ^18^F-FES study in humans without neurological disorders was conducted on de novo breast cancer patients, showing a non-significant trend toward higher pituitary uptake in postmenopausal as compared to premenopausal patients^23^. However, the study had an uneven sample size, with fewer premenopausal (n = 9) compared to postmenopausal women (n = 22), and PET imaging in the premenopausal group was not standardized to the menstrual cycle^23^, potentially reducing power to detect significant differences. Additionally, no age correction procedures were applied to control for the wide participant age range (23–76 years, median 57)^23^. Technical limitations included pituitary delineation based solely on tracer uptake rather than MRI-guided tracing; measurement of ^18^F-FES uptake using standardized uptake values (SUVs) rather than kinetic modeling; and normalization of pituitary signal to that in frontal cortex^23^—a known ER site^28–31^—which may have further hindered detection of menopausal effects.

The present brain ^18^F-FES PET study examined a prospective cohort of healthy midlife women ages 40–65 years, divided into three size-matched groups based on menopausal status. All postmenopausal women had undergone menopause spontaneously, and all premenopausal participants were scanned around midcycle (Methods). We used kinetic modeling and state-of-the-art ROI analysis to examine ^18^F-FES data using an anatomically and statistically-validated cerebellar reference region developed via supervised clustering algorithms (Methods). Using these procedures, our results show a pattern of brain ER distribution which maps onto estrogen-regulated neural systems^3^, and is consistent with preclinical *ex vivo*^28–31^ and in vivo work^22,23^. In line with the above breast cancer study^23^, the pituitary gland showed the strongest tracer uptake, which was highest in postmenopause, intermediate in perimenopause, and lowest in the premenopausal group. The other regions also exhibited progressively higher ER density from premenopausal to postmenopausal stages. Interestingly, ER density in PCC was elevated at the perimenopausal stage relative to premenopausal controls, suggesting an early role for this region in the neuroendocrine aging process. Also of interest was the lack of interactions of age and menopause status. While some age effects were observed, there were additional independent effects of menopause status on ER density, which were not modified by age. Overall, these findings provide novel evidence for associations between female neuroendocrine aging and progressively higher ER density in E2-regulated brain areas involved in various reproductive and higher-cognitive functions^1,2^.

Our exploratory voxel-based analysis revealed some asymmetry in ER density associated with menopause status, with more consistent effects in the left hemisphere. At a more liberal *P* < 0.001, additional clusters of higher DVR postmenopause emerged, including insula, anterior cingulate, precentral and parietal cortex, predominantly in the left hemisphere. This laterality may reflect underlying differences in hemispheric dominance for cognitive and emotional processes that are susceptible to estrogen's modulatory action and may respond differently to estrogen depletion^35^. Additionally, hemispheric variations in cerebral blood flow or metabolic activity, processes which are impacted by neuroendocrine aging^10–12^, could contribute to the observed patterns. Future studies with larger samples are needed to replicate these findings and explore possible sources of asymmetry in ^18^F-FES uptake.

Factors contributing to the neurological symptoms of menopause have not been clearly identified. While declines in circulating sex hormones, chiefly E2, are assumed to account for these symptoms^8^, direct evidence is lacking. Hence, a novel finding of this study is the evidence for associations of ER density with presence of current menopausal symptoms of neurological origin, chiefly mood changes and memory complaints. Increasing ER levels in estrogen-regulated amygdala, PCC and frontal regions were associated with a higher likelihood of experiencing these symptoms, providing a neurophysiological basis for clinical observations. Herein, we did not observe significant associations between ^18^F-FES signal and presence of vasomotor symptoms, which have been linked to the function of the hypothalamus^1–3^. Other studies are needed to investigate whether physiologically monitored hot flashes, rather than self-reported presence of hot flashes, would exhibit significant associations. Considering the hypothalamus’s central role in neuroendocrine function and menopausal symptomatology, further studies are warranted to explore ER expression in this region.

The extent to which ovarian steroids impact cognitive function in menopause also remains controversial^36–38^. Our study provides new evidence for significant associations between brain ER density and memory function in women of menopausal age. In analysis of standardized cognitive tests, higher ER density in hippocampus was associated with worse memory performance for both postmenopausal and perimenopausal groups. Additionally, higher ER density in amygdala, PCC and frontal cortex correlated with lower memory scores in the postmenopausal group. As all participants scored within normal limits for age and education, these findings align with observations that, while menopause itself isn’t linked to cognitive deficits, subjective memory complaints are common in women of menopausal age and reflect subtle but measurable changes in performance^35,36^.

Estrogen therapy with or without progesterone is the recommended treatment for menopausal hot flashes. It also holds promise for support of cognitive function^39,40^ and for attenuation of neuronal injury and cell death resulting from neurodegenerative insults^9,18,41,42^, especially when started in midlife, but not once neurological disease is established^40,43^. Therefore, as women approach menopause, there seems to be a critical window of opportunity not only to detect signs of neurological risk but to intercede with strategies to reduce or prevent that risk by ameliorating estrogen levels^44^. While long-term E2 deprivation has been shown to lead to degradation of ERα in some regions^45^, present neuroimaging findings indicate that the brain may be undergoing estrogenic adjustments in postmenopausal women up to the age of 65 years. These results may help home in on the window of opportunity for therapeutical intervention, and provide biological markers of neurological vulnerability for future examinations of estrogen therapy.

### Technical considerations

Higher ^18^F-FES binding postmenopause may be due to either the near-absence of endogenous E2 competing for receptor occupancy, resulting in reduced tracer dilution, or to an E2 depletion-induced positive feedback loop triggering ER upregulation as a compensatory response aimed at preserving neural function in E2-reliant regions. While possible dilution reduction effects cannot be fully excluded, several lines of evidence suggest presence of compensatory mechanisms. First, ^18^F-FES studies in rodents have shown a 1.7-fold increase in brain ER density following bilateral oophorectomy^22,23^, suggesting that increased tracer binding in postmenopausal women could also be attributed to increased ER density, rather than reduced competition from circulating E2. Furthermore, rodent studies indicate that brain ER expression is in part inversely associated to E2 levels during the menstrual cycle^46^, strengthening the argument for enhanced ER expression following the permanent decline in E2 after menopause. Additionally, preclinical research has shown that, while circulating E2 levels may influence delivery of ^18^F-FES to the brain, its binding to brain ERs is less or not impacted^22^. Hormonal differences between female rats and women notwithstanding, studies of breast cancer patients have similarly reported mild or non-significant impacts of circulating E2 on ^18^F-FES uptake in peripheral tissues^47^. In this study, the observed group differences in ER binding were significant after adjusting for plasma E2 levels, supporting evidence of independent effects in brain versus periphery.

While E2 can regulate its own receptor gene expression in the adult brain^47^, other factors control ERα mRNA in addition to circulating levels^48^. For instance, E2 is mainly produced in the ovary, but it is also locally synthesized in brain^1,2^. Brain steroidogenesis is regulated independently of peripheral steroidogenesis, and plasma steroid levels do not directly reflect brain steroid levels^4^, which may have contributed to our findings. Further, after oophorectomy in female rats, the brain appears to adapt the levels of steroid synthesis as a compensatory adaptation of brain steroidogenesis in response to gonadal steroid deprivation^49^. Although aromatase activity is reduced with aging and menopause^50^, the brain retains the ability to synthesize E2 locally under conditions of neurological stress^4^. In response to brain injury and stroke, endogenous E2 synthesis and ER expression are both upregulated in neurons, and de novo synthesis is induced in astrocytes, independent of ovarian production^4^. It is possible that neurological stress during the menopause transition might trigger similar responses. Additionally, estrone (E3, the most prevalent type of estrogen post-menopause) can both bind to ERα and be converted into E2. This may further justify the need for an increase in ER density. Overall, elevations in brain ER density independent of menopause-related plasma E2 fluctuations are in line with a compensatory neurophysiological response to the loss of ovarian hormones.

### Strengths and limitations

To our knowledge, this is the first in vivo brain imaging study to investigate ER modulation by neuroendocrine aging in humans. We focused on carefully screened, healthy midlife women ages 40–65 years, with complete clinical and cognitive exams, laboratory tests, menopause assessments, and brain imaging. Our extensive exclusion criteria ensured absence of confounding factors such as cancer, oophorectomy / hysterectomy, and HT use. From a methodological perspective, we examined women at different menopausal stages, paired with age correction procedures, as a natural experiment of estrogen decline. We used a combination of state-of-the-art ROI and voxel-based analysis while taking a translational approach to ensure that our results were both statistically and biologically valid. Results were significant after multivariable correction for age, modality-specific confounders, and plasma E2 and SHBG levels measured shortly prior to imaging.

We chose a cross-sectional design because the timing of menopause is highly variable, with a median age at menopause of 51 years and distribution 40–58 years^8^. Longitudinal studies may require 10–15 years of follow-ups to capture changes in brain ERs in the same women. While oophorectomy ideally reduces follow-up times, the procedure yields different neurophysiological outcomes^7,8^. Nonetheless, given the cross-sectional and observational nature of this study, a temporal and causal relationships between exposures and outcomes cannot be unequivocally established. Longitudinal studies are warranted to characterize the temporal trajectories of ER changes in relation to neuroendocrine aging, and to test for differences between induced and spontaneous menopause. We caution that present results are obtained from small samples of carefully screened research participants with at least 12 years of education. Replication in community-based populations with diverse educational, racial or socio-economical background is warranted. Further research is needed to explore the influence of genetic factors, such as genetic polymorphisms affecting ER expression, as well as the roles of stress and other social factors on brain ^18^F-FES uptake.

Kinetic modeling with absolute quantification remains the gold-standard for PET neuroreceptor studies. Absolute quantification is however invasive due to the need for continuous arterial blood sampling, while also being subject to error-prone metabolite analysis^51^. Herein, we used graphic reference-tissue Logan plots^27^ to derive ^18^F-FES DVRs, using a custom-built cerebellar gray matter region as the reference. As described in the Methods, this choice was based on preclinical ex vivo and in vivo evidence that cerebellar crus II gray matter generally does not express, or minimally expresses, ERα^28–31,52^. The ^18^F-FES signal predominantly originates from ERα-expressing areas^19^, with the tracer exhibiting six times greater binding affinity for ERα than for ERβ^53^. Although the specific binding affinities of ^18^F-FES to ER subtypes within the human brain remain uncharacterized, and our in vivo imaging techniques do not permit estimation of relative ER subtypes densities, preclinical studies indicate that in the adult rat cerebellum, ERβ expression exceeds that of ERα by more than 50-fold^54^. Moreover, our cerebellar reference ROI was further optimized by means of supervised clustering algorithms (SCA) to ensure that tracer uptake was both low and invariant by exposure (e.g. menopause status), which is a key prerequisite for normalization^55^. SCA methods are commonly used to extract pseudo-reference regions for non-invasive quantification of PET tracers with low intracerebral uptake, such as activated microglia TSPO (translocator protein) ligands^56^. In female rodents, kinetic ^18^F-FES analyses show a nearly 70% increase in pituitary ER binding after oophorectomy^22,23^. In our study, the postmenopausal group exhibited 36% higher DVRs in pituitary compared to the premenopausal group, which seems physiologically plausible for women undergoing spontaneous menopause. Nonetheless, it is possible that we may have underestimated ER density in the cerebellar ROI. This would however affect all menopausal groups, thus conservatively reducing power in detecting group effects. Given the high anatomical agreement between the observed ER distribution and preclinical literature; the robust effect size resulting from group comparisons; and the coherence of correlational patterns with cognition and menopausal symptomatology, we attribute our results to cerebral ER expression in response to ovarian declines in E2 production during midlife female neuroendocrine aging.

A main limitation of the ^18^F-FES ligand, partly due to its high lipophilicity, is its non-specific binding. Specifically, previous research indicates that tracer uptake in white matter could not fully blocked by the administration of cold tracer^25,26^, thus reflecting non-specific uptake. Although white matter regions were excluded from the current analysis, visual inspection revealed more pronounced white matter uptake in the postmenopausal group compared to the premenopausal group, as exemplified in Fig. 1D. Given that ERs are expressed in glial cells and oligodendrocytes within white matter^2,3^ and that menopause has been associated with white matter changes^10,12,15^, further studies are warranted to discern specific from non-specific binding in these areas. Novel ER ligands with lower nonspecific signal and likely better contrast than ^18^F-FES PET are also being developed, such as 4-fluoro-11β-methoxy-16α-^18^F-fluoroestradiol (^18^F-4FMFES)^23^. Additionally, PET tracers with higher selectivity for ERβ, such as 2-[^18^F]-fluoro-6-(6-hydroxynaphthalen-2-yl)pyridin-3-ol (^18^F-FHNP), as well as progesterone receptor (PRs) ligands such as ^18^F-fluoro-furanyl-norprogesterone (^18^F-FFNP) are of considerable interest.

Overall, because ERs mediate estrogen actions, and changes in ER expression themselves participate in cognitive function, mental health and disease development, ER-PET imaging represents an essential advance in our understanding of sex hormones’ impact in brain—while also opening the possibility of using ER ligands to monitor the efficacy of estrogen treatment in clinical trials and clinical practice.

## Conclusion

This proof-of-concept study provides novel insights on brain ER density modulation by female neuroendocrine aging, with clinical implications for women’s health. Findings provide a neurophysiological substrate for the neurological vulnerability observed in menopausal women and for the posited ‘window of opportunity’ for preventative strategies.

## Methods

### Participants

This is a natural history, non-interventional study of 54 consecutive clinically and cognitively normal midlife women at different endocrine stages, including equal proportions of premenopausal (standardized to midcycle), perimenopausal, and postmenopausal participants. Participants were recruited at Weill Cornell Medicine (WCM) between 2021 and 2024 from multiple community sources, including individuals interested in research participation, family members and caregivers of impaired patients at our institution, and by word of mouth^10–12,15^.

All participants gave written informed consent to participate in this ^18^F-fluoroestradiol (^18^F-FES) positron emission tomography (PET) study, which was approved by the WCM Institutional Review Board. Use of ^18^F-FES was carried out under WCM Radioactive Drug Research Committee and National Cancer Institute (NCI) Investigational New Drug (IND) #146703 approval. All experiments were performed in accordance with relevant guidelines and regulations.

Participants were 40–65 year-old women with ≥ 12 years of education and a diagnosis of normal cognition per physician’s assessment, with Montreal Cognitive Assessment (MoCA) scores ≥ 26 and cognitive test performance within normative values for age and education^10–12,15^. Pre-established exclusion criteria included: (i) any significant neurological disease, such as dementia, normal pressure hydrocephalus, brain tumor, progressive supranuclear palsy, seizure disorder, subdural hematoma, multiple sclerosis, or history of significant head trauma followed by persistent neurologic deficits or known structural brain abnormalities; (ii) any significant psychiatric disease, such as major depression, bipolar disorder, schizophrenia, or psychotic features; (iii) T2 and/or FLAIR MRI brain scan evidence of infarction, lacunes or demyelination disease; (iii) systemic illnesses, unstable medical conditions or major medical complications such as treatment for neoplastic disease, unmanaged cardiovascular disease, diabetes, renal or liver disorder; (iv) history of drug or alcohol dependence; (v) current use of psychoactive medications (e.g. benzodiazepines, cholinesterase inhibitors, psychostimulants, etc.) or investigational agents; (vi) contraindications to MRI or PET imaging. Additional exclusion criteria included: (vii) history of oophorectomy or hysterectomy; (viii) use of hormonal therapy including oral contraceptives and menopause hormone therapy; (ix) active pregnancy.

All participants underwent clinical examinations including medical history, neurological exams, neuropsychological testing, blood analysis including genetics and sex steroid hormones, volumetric MRI and ^18^F-FES PET scans. The patients’ sex was determined by self-report. Participants were enrolled into three size-matched groups according to menopausal status based on the Stages of Reproductive Aging Workshop (STRAW)^57^ with hormone laboratory assessments as supportive criteria (premenopause: no change in menstrual regularity in the past 12 months; perimenopause: irregular cycle, no menses in the past 3–11 months; postmenopause: no menses for the past ≥ 12 months)^57^. Participants were therefore not randomly assigned to groups. All participants were asked to report the date of their last two menstrual periods for diagnostic purposes. PET studies of premenopausal participants were scheduled to coincide with the next nearest midcycle, when plasma E2 levels are highest. To determine the appropriate timing, we calculated the cycle length based on the participant’s last two menstrual periods, and midcycle was identified as the time approximately halfway through the cycle, specifically the week around ovulation, which typically occurs around day 14 in a 28-day cycle. Cycle irregularities in perimenopausal women prevented scheduling their PET scans according to a specific menstrual cycle phase. However, plasma E2 and SHBG were collected from all participants and examined as covariates.

Sex steroid hormone levels were measured by a CLIA-certified commercial laboratory (Boston Heart Diagnostics, Framingham, MA). E2 and SHBG were assessed through competitive immunoassay with a measuring range of 5–3000 pg/mL and 0.8–200 nmol/L. Blood samples were taken on the day of the PET study for all participants except two who did the blood draw the day prior. To test whether tracer binding was impacted by competition with endogenous E2, we included E2 as a covariate, which only enhanced differences in ER density between menopausal groups. Including SHBG as a covariate had similar effects. All results presented in the manuscript are adjusted by both E2 and SHBG.

Participants completed the Menopause Health Questionnaire^58^ and the Menopause Rating Scale^59^ for evaluation of symptom clusters including presence of vasomotor symptoms and menopausal-related changes in mood, sleep, libido, and cognition, relative to the premenopausal stage^10–12,15^. For this study, participants were asked to self-report whether they were also currently experiencing these symptoms, referring to any occurrences within the past month leading up to the imaging session, and to commit to a binary “yes/no” response. Symptom presence was examined as a correlational outcome.

A total of 60 participants were enrolled. Three were excluded prior to PET imaging, of whom 2 due to conditions encountered in the MRI scan (demyelination) and 1 participant with a positive pregnancy test. Additionally, we excluded 1 premenopausal participant who was imaged during the luteal phase, and 2 participants due to technical reasons (motion artifacts). Our final study cohort included 54 consecutive participants, divided into three groups of 18 each, according to menopause status. Three participants with hypertension were under medical management and had stable conditions.

### Cognitive testing

We focused on tests with known sensitivity to estrogen levels^12,36,40^, including verbal memory [immediate and delayed recall of Rey Auditory Verbal Learning Test (RAVLT) and Wechsler Memory Scale logical memory tests], executive function (Trail Making Test B), fluency (FAS and animals), and language (object naming) tests.

### Brain imaging

#### Acquisition

All participants received MRI and PET scans following standardized protocols^10–12,15^. Scans were performed on consecutive days, except for 9 participants who completed FES an average of 0.8 ± 1.9 months before or after MRI. Adjusting by time between scans as a covariate did not significantly impact the results.

*Volumetric MRI*. 3D volumetric T_1_-weighted MRI scans [BRAVO; 1 × 1 × 1 mm resolution, 8.2 ms repetition time (TR), 3.2 ms echo time (TE), 12° flip angle, 25.6 cm field of view (FOV), 256 × 256 matrix with ARC acceleration] were acquired on a 3.0 T MR750 Discovery scanner (General Electric, Waukesha, WI) using a 32-channel head coil.

^*18*^*F-Fluoroestradiol PET imaging*. 16α-[^18^F]fluoro-17β-estradiol (^18^F-FES) was prepared by the WCM PET Radiochemistry Group using established methods for synthesis and quality assurance^60,61^ [https://imaging.cancer.gov/programs_resources/cancer-tracer-synthesis-resources/FES_documentation.htm]. ^18^F-FES scans were acquired using a Siemens BioGraph mCT 64-slice PET/CT scanner [70 cm transverse FOV, 16.2 cm axial FOV, voxel size 1.0 mm] operating in 3D mode. All scans were performed after a 4-h fasting to decrease biliary uptake. One hour before PET imaging, an antecubital venous catheter was positioned for tracer injection. No arterial blood sampling was performed. Participants lied down on the scanner bed with eyes closed and ears unplugged, in the quiet and dimly lit scan room. Following a low-dose CT scan, a dose of approximately 6 mCi (222 MBq) of ^18^F-FES was infused intravenously in a volume of 20 mL isotonic phosphate buffered saline containing less than 15% of ethanol by volume over 2 min. Dynamic imaging was performed for 90 min, and consisting of 30 frames: 4 × 15, 4 × 30, 3 × 60, 2 × 120, 5 × 240, 12 × 300 s. All images were corrected for attenuation, scatter and radioactive decay.

### Image analysis

Image processing was performed using a semi-automated pipeline^10–12,15^. The T1-weighted images were first segmented using SPM Segment and spatially normalized by high-dimensional warping (DARTEL) implemented in SPM12^33^ running on Matlab 2021 (MathWorks; Natick.MA). ^18^F-FES dynamic images were motion-corrected by first creating a mean image of early 1–8 min frames, and then using that as an anchor for all frames within-modality and for co-registration to the T1-weighted image, using the surface-fitting Normalized Mutual Information (NMI) algorithm^33^. The spatial transformation from the DARTEL operation was then applied to all motion-corrected co-registered ^18^F-FES frames (affine transformation using 5^th^ degree B-spline interpolation, final voxel size 1.5 × 1.5 × 1.5 mm).

*Target regions*. While ERs are widely expressed throughout the brain, their density varies by isoform and region^1,2^. As ^18^F-FES selectively binds ERα^53^, and tracer uptake in white matter is affected by non-specific binding^25,26^, we focused on predominantly gray matter regions with high ERα expression. These included pituitary, hypothalamus, thalamus, hippocampus, amygdala, caudate, posterior cingulate (PCC), middle and inferior frontal cortex^28–31^. The pituitary ROI was manually delineated on the coregistered anatomical MRI by three expert raters (DM, MN, VB) according to published criteria^62^.

*Reference region*. We chose the cerebellum as the anatomical reference region based on evidence that it is generally void of ERα^29–32,51^. We then developed a probabilistic cluster-based cerebellar ROI, using the following procedures, illustrated in Supplementary Fig. 1: (i) given evidence that ERβ and GPER-1 are expressed in the innermost portion of cerebellar white matter and adjacent gray matter (corresponding to human middle cerebellar peduncle, culmen, arbor vitae, dentate nucleus, and medullary cortex)^29–32^, the cerebellar ROI was also restricted to the outermost portion of cerebellar crus II gray matter, which is generally free of ERs; (ii) voxel-based machine learning with intensive iterative data resampling implemented in NPAIRS (nonparametric prediction, activation, influence, and reproducibility resampling)^63^ was used to further restrict the ROI to the inferior portion of cerebellar crus II, which showed invariant tracer uptake across menopause classes. Supplementary Fig. 1B illustrates the final reference region.

*Distribution volume and parametric image generation*. ROI placement, thresholding, and sampling were conducted with PMOD v4.1 (PMOD Technologies). ^18^F-FES images were quantified using select ROIs from the anatomical labeling atlas (AAL3)^64^ implemented in PMOD v4.1, applied to each participant’s PET. ROIs were applied to motion-corrected dynamic PET images to obtain regional TACs of tissue radioactivity concentration across all slices sampled. Graphic Logan plots were used to estimate distribution volume ratios (DVR) (1 + BP, binding potential) in each target region using the TAC of the cerebellar reference ROI as the reference^27^. Parametric BP images were also generated using PMOD pixel-wise modeling tool (PXMOD) using the cerebellar TAC as the reference. Only voxels with BP > 0 were retained. For voxel based analysis, MRI scans were spatially normalized to the template-normalized tissue probabilistic map (TPM) image included in SPM12 conforming to the MNI space and filtered with an 8 mm full-width at half maximum (FWHM) smoothing kernel^33^. The MRI-coregistered parametric ^18^F-FES BP images were then spatially normalized to the TPM image using MRI-derived subject-specific transformation matrices and smoothed at 8-mm FWHM^33^.

### Statistical analysis

Analyses were performed in SPSS v.25, R v.4.2.0 and SPM12. Clinical measures were examined with general linear models or chi-squared tests as appropriate. All brain image analyses are adjusted by age (years), plasma E2 (pg/mL) and SHBG (nmol/L).

### ER density by menopause status

Multivariable linear regression models were trained to consider the effect of a three-level exposure variable (levels: pre, peri, and postmenopause status) on ^18^F-FES DVR across all target ROIs. Regression models were constructed to obtain global P values for multivariate pair-wise outcomes through Games-Howell tests, at *P* < 0.05. For models showing significant main effects, differences between groups were explored using forest plots for assessment of individual regions.

### Prediction of menopause status

ROI DVRs were examined for menopause status separation using Cohen’s d effect size. After adjustment by confounders, the standardized pairwise mean differences between any two levels were expressed as Cohen’s d coefficients, where d ≥ 0.8 reflects a large effect size. We used a conservative cut-off of 1.5 to identify the regions yielding the largest effect size in separating groups. To gauge the degree to which ER density in these regions was predictive of menopause status, predictive models by means of multivariable logistic regressions were trained on a random 80% of the study sample, with 20% withheld as the testing set. Each model contained the binary outcome, premenopause vs. postmenopause status. Our primary outcome was the percent accuracy in the testing set, defined as the proportion of correct predictions over total predictions. Global likelihood ratio tests were performed for each model at *P* < 0.05.

### Associations of ER density and cognitive performance

To test for associations between ER density and cognitive scores, we developed linear regression models with standardized cognitive test scores as the outcomes, and ER density as the exposure of interest, adjusting for the above confounders, at *P* < 0.05. To limit the number of comparisons, hypothesis testing was restricted to one test per cognitive domain (logical memory, TMT-B, object naming, and animal naming) and brain regions with established cognitive functions: hippocampus (memory formation, learning, encoding and retrieval of verbal information), PCC (episodic memory and visual processing) and frontal regions (executive function, language and verbal fluency)^65^. Analysis was performed across all participants and separately for postmenopausal and perimenopausal groups. The variance in cognitive scores within the premenopausal group was insufficiently broad to enable meaningful assessment of correlations with ^18^F-FES data.

### Associations of ER density and menopause symptoms

To test for associations between ER density and menopause symptoms, we developed logistic regression models with menopause symptom occurrence as the binary outcome variable, and ER density as the exposure of interest, adjusting for confounders, at *P* < 0.05. Analyses were performed for perimenopausal and postmenopausal groups combined, and separately. Odds ratios (OR) were estimated, where a positive value denotes a positive association between ER density and presence of each menopause symptom. OR that exceeded 10 were capped at 10 to ensure visibility of positive associations < 10 in the figure.

### Sensitivity analysis

#### Voxel-wise analysis of ER density by menopause status

In addition to the ROI analysis, we conducted a voxel-based analysis of parametric ^18^F-FES BP images using factorial models with post-hoc *t*-contrasts as implemented in SPM12^33^ to test for differences between menopause groups within and outside of pre-selected ROIs, adjusting for the above confounders. Statistical maps were constructed by applying a voxel-level Gaussian random field theory–based threshold of *P* < 0.05, cluster-level corrected for Family-Wise Type Error (FWE) within a binary masking image consisting of the entire frontal, temporal, parietal and cingulate gray matter. Only clusters ≥ 16 voxels were considered significant (e.g., twice the FWHM) to further reduce the likelihood of Type I errors^33^. For completeness, we also examined results at a more liberal uncorrected threshold of *P* < 0.001 within the SPM12 whole-brain gray matter image. Anatomical location of significant clusters was described using Talairach coordinates after conversion from MNI space.

#### Effects of age, and age by menopause status interactions on ER density

Given the expected age difference between the premenopausal and postmenopausal groups, the following published procedures were applied to differentiate the effects of age and menopause status on the outcomes^12,32^: (i) we used box plots and frequency diagrams to ensure that we had sufficient age overlap among different menopause statuses, which enabled us to test for effects of endocrine aging separately from chronological aging; (ii) age was evaluated as a covariate in all analyses; (iii) multivariable linear regression models were trained to test for associations between age and ROI DVR measures, adjusting by clinical confounders. Estimates are presented for the overall study sample as well as for each menopausal group. This stratified analysis was performed to investigate hypothesized differences in the strength of age-ER density correlations by menopause status; and (iv) we tested for age-based modification effects by developing linear regression models including age, menopause status, and their interaction as covariates, at *P* < 0.05.

### Supplementary Information

Supplementary Figure 1.

Supplementary Tables.

## Data Availability

De-identified source data files and statistical code will be made available to qualified investigators for the purpose of replicating procedures and results upon reasonable request to the corresponding author.
