# Supplementary material for: In vivo brain estrogen receptor density by neuroendocrine aging and relationships with cognition and symptomatology
Source: Sci Rep. 2024 Jun 20;14:12680. doi: 10.1038/s41598-024-62820-7 (PMC11190148; doi:10.1038/s41598-024-62820-7)
Supplement: Supplementary file 1 — Supplementary Figure 1. [file 41598_2024_62820_MOESM1_ESM.docx]

## Supplementary Figure 1. Development of the cerebellar reference region

##
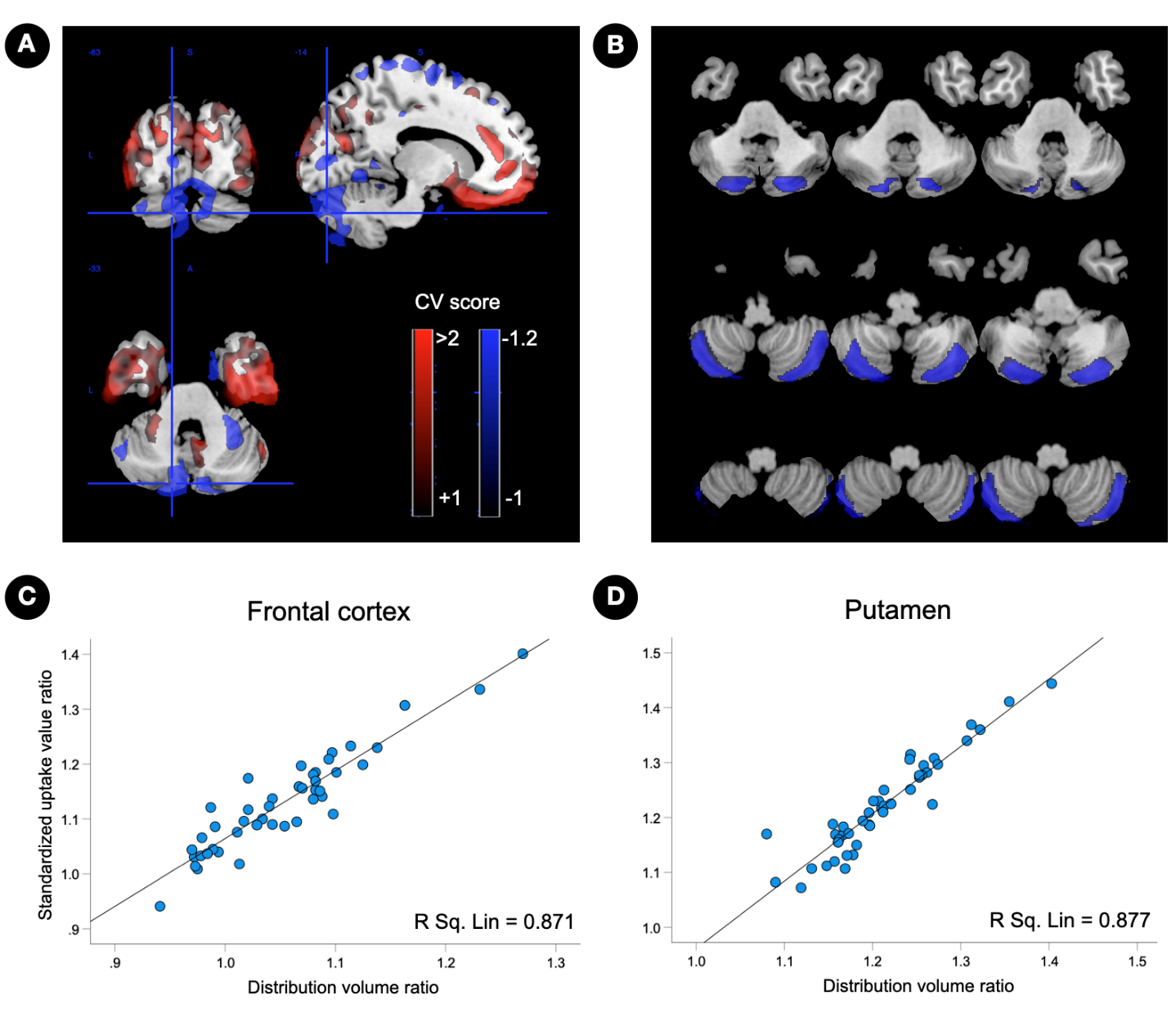


Multivariate voxel-based Canonical Variates (CV) analysis, e.g. linear discriminant analysis, was performed in NPAIRS (nonparametric prediction, activation, influence, and reproducibility resampling) to optimize the cerebellar reference region-of-interest (ROI). To achieve this, NPAIRS was trained on ^18^F-FES PET images to produce a set of image intensity patterns that best discriminated between pre-, peri-, and post-menopausal classes. In NPAIRS, the patterns that differentiate classes show both relative multivariate increases and decreases in uptake, as well as no differences. This can be used to identify potential reference regions, e.g. clusters that are opposite to or independent of target signal, therefore offering the greatest inter-class discrimination for reference normalization.

**(A)** The pattern of voxel intensities that is increasingly expressed by menopause stage as identified by NPAIRS is shown, where voxels in red represent increasing ^18^F-FES uptake, and those in blue represent preserved or decreased intensity (e.g., a lack of differences in ^18^F-FES uptake) relative to whole brain. CV scores are displayed using color-coded scales with positive (red) and negative (blue) values, respectively. These regions correspond to those reported in the literature as having greater and lower levels of ER-alpha, respectively. Specifically for the cerebellum, NPAIRS identified tissue including the inferior portion of cerebellar crus II gray matter as a suitable reference region for normalization of ^18^F-FES data, as this portion of the cerebellum exhibited the lowest ER binding and relative invariance across menopause statuses. Both are pre-requisites for data normalization.

**(B)** NPAIRS was then used to identify and distance the cerebellar ROI boundaries from locations where on-target and/or off-target uptake were prominent. The reference region was thus refined by slight erosion of voxels from superior, anterior and inferior slice voxels from the boundaries with neighboring regions: (i) superior voxels were removed to increase distance from occipital cortex where signal spill-in could occur; (ii) anterior voxels were removed to avoid cerebellar white matter as well as portions of cerebellar cortex where inter-subject variability is greater; and (iii) inferior slices were removed due to technical variability that occurs in those slices regardless of PET tracer. Panel **(B)** illustrates the final reference region overlaid on a MRI template tissue probability map. The reduction in volume of the reference region did not increase signal variability, and values were similar but slightly reduced in the most eroded version. This suggests a reduction in signal spill-in or inclusion of voxels affected by the presence of ERs. This reference region was used to account for inter-individual variability in ^18^F-FES uptake in subsequent analyses.
