# Supplementary material for: In vivo brain estrogen receptor density by neuroendocrine aging and relationships with cognition and symptomatology
Source: Sci Rep. 2024 Jun 20;14:12680. doi: 10.1038/s41598-024-62820-7 (PMC11190148; doi:10.1038/s41598-024-62820-7)
Supplement: Supplementary file 2 — Supplementary Tables. [file 41598_2024_62820_MOESM2_ESM.docx]

## Supplementary Table 1. Voxel-wise effects of menopause status on brain estrogen receptor density

| Cluster extent | Coordinates  x,y,z | Z | P_FWE_* | P voxel | Anatomical Region |
| --- | --- | --- | --- | --- | --- |
| **Postmenopause > Perimenopause > Premenopause** | | | | | |
| 485 | -15 36 -8 | 3.62 | 0.004 | <0.001 | Anterior Cingulate Cortex, Left |
|  | -9 22 -4 | 3.45 |  | <0.001 | Caudate Nucleus, Left |
|  | -8 20 12 | 3.28 |  | <0.001 | Caudate Nucleus, Left |
| 94 | -36 27 33 | 3.59 | 0.030 | <0.001 | Middle Frontal Gyrus, Left |
| 51 | 16 40 38 | 3.55 | 0.041 | <0.001 | Superior Frontal Gyrus, Right |
| 136 | 42 42 4 | 3.52 | 0.023 | <0.001 | Middle Frontal Gyrus, Right |
| 54 | -50 -45 38 | 3.39 | 0.040 | <0.001 | Inferior Parietal Lobule, Left |
| 47 | 16 58 15 | 3.26 | 0.043 | <0.001 | Superior Frontal Gyrus, Right |
| **Postmenopause > Premenopause** | | | | | |
| 111 | 16 40 38 | 3.78 | 0.041 | <0.001 | Superior Frontal Gyrus, Right |
| 862 | -9 21 -6 | 3.61 | 0.002 | <0.001 | Caudate Nucleus, Left |
|  | -15 36 -8 | 3.53 |  | <0.001 | Anterior Cingulate Cortex, Left |
|  | -8 20 12 | 3.51 |  | <0.001 | Caudate Nucleus, Left |
| 213 | 42 42 4 | 3.57 | 0.023 | <0.001 | Middle Frontal Gyrus, Right |
|  | 34 48 0 | 3.41 |  | <0.001 | Middle Frontal Gyrus, Right |
| 110 | -21 -3 24 | 3.47 | 0.041 | <0.001 | Caudate Nucleus, Left |
|  | -16 -14 26 | 3.25 |  | <0.001 | Caudate Nucleus, Left |
| 107 | 16 60 14 | 3.39 | 0.042 | <0.001 | Superior Frontal Gyrus, Right |
| **Postmenopause > Perimenopause** | | | | | |
| 167 | 40 30 20 | 4.24 | 0.020 | <0.001 | Middle Frontal Gyrus, Right |
| 249 | 36 54 2 | 3.71 | 0.013 | <0.001 | Middle Frontal Gyrus, Right |
| 221 | -48 51 8 | 3.57 | 0.015 | <0.001 | Middle Frontal Gyrus, Left |
| 88 | 18 40 38 | 3.53 | 0.033 | <0.001 | Superior Frontal Gyrus, Right |
| 183 | -21 -4 24 | 3.47 | 0.018 | <0.001 | Caudate Nucleus, Left |
|  | -8 4 21 | 3.37 |  | <0.001 | Caudate Nucleus, Left |
| 140 | -4 18 10 | 3.37 | 0.024 | <0.001 | Caudate Nucleus, Left |
|  | 0 14 0 | 3.22 |  | <0.001 | Caudate Nucleus, Left |
| 52 | -27 52 -3 | 3.26 | 0.043 | <0.001 | Superior Frontal Gyrus, Left |
|  | -33 62 2 | 3.10 |  | <0.001 | Superior Frontal Gyrus, Left |
| **Perimenopause > Premenopause** | | | | | |
| 63 | -30 26 33 | 3.52 | 0.004 | <0.001 | Superior Frontal Gyrus, Left |
| 18 | -45 -44 38 | 3.22 | 0.006 | <0.001 | Inferior Parietal Lobule, Left |

**P* < 0.05 cluster-level corrected for multiple comparisons within the search volume. Analyses are adjusted by age, plasma estradiol and sex hormone binding globulin levels.

## Supplementary Table 2. Exploratory voxel-wise analysis of menopause status on brain estrogen receptor density at an uncorrected threshold of P<0.001

| Cluster extent | Coordinates  x,y,z | Z | P* | Anatomical Region |
| --- | --- | --- | --- | --- |
| **Postmenopause > Perimenopause > Premenopause** | | | | |
| 278 | -36 27 32 | 3.63 | <0.001 | Middle frontal gyrus, left |
|  | -28 24 28 | 3.57 | <0.001 | Middle frontal gyrus, left |
|  | -26 16 21 | 3.22 | <0.001 | Insula, left |
| 333 | -15 36 -8 | 3.57 | <0.001 | Anterior cingulate cortex, left |
|  | -9 21 -6 | 3.31 | <0.001 | Caudate, left |
|  | -8 28 6 | 3.23 | <0.001 | Anterior cingulate cortex, left |
| 47 | 16 40 38 | 3.56 | <0.001 | Superior frontal gyrus, right |
| 118 | -32 3 42 | 3.54 | <0.001 | Precentral gyrus, left |
| 178 | 40 42 4 | 3.48 | <0.001 | Middle frontal gyrus, right |
|  | 34 46 -2 | 3.19 | <0.001 | Middle frontal gyrus, right |
| 47 | -48 -45 38 | 3.36 | <0.001 | Inferior parietal lobule, left |
| 22 | -12 27 34 | 3.31 | <0.001 | Superior frontal gyrus, left |
| 33 | 16 58 15 | 3.25 | <0.001 | Superior frontal gyrus, right |
| 22 | -22 -2 22 | 3.20 | <0.001 | Caudate, left |
| **Postmenopause > Premenopause** | | | | |
| 104 | 16 40 38 | 3.80 | <0.001 | Superior frontal gyrus, right |
| 143 | -32 3 42 | 3.57 | <0.001 | Precentral gyrus, left |
| 204 | -26 16 21 | 3.28 | <0.001 | Insula, left |
|  | -36 27 32 | 3.54 | <0.001 | Middle frontal gyrus, left |
| 278 | 40 40 4 | 3.54 | <0.001 | Middle frontal gyrus, right |
|  | 33 46 -2 | 3.43 | <0.001 | Middle frontal gyrus, right |
| 297 | -21 -3 22 | 3.51 | <0.001 | Caudate, left |
|  | -18 -14 28 | 3.32 | <0.001 | Caudate, left |
| 193 | -15 36 -8 | 3.49 | <0.001 | Anterior cingulate cortex, left |
|  | -15 51 0 | 3.20 | <0.001 | Superior frontal gyrus, left |
| 258 | -9 21 -6 | 3.48 | <0.001 | Caudate, left |
|  | -8 26 10 | 3.38 | <0.001 | Anterior cingulate cortex, left |
| 76 | 16 58 15 | 3.40 | <0.001 | Superior frontal gyrus, right |
| 33 | -50 -45 38 | 3.34 | <0.001 | Inferior parietal lobule, left |
| 23 | -12 27 34 | 3.32 | <0.001 | Superior frontal gyrus, left |
| 62 | -40 40 6 | 3.29 | <0.001 | Inferior frontal gyrus, left |
| 34 | -8 4 24 | 3.24 | <0.001 | Anterior cingulate cortex, left |
| **Postmenopause > Perimenopause** | | | | |
| 232 | 40 30 20 | 4.21 | <0.001 | Middle frontal gyrus, right |
| 185 | 36 54 2 | 3.67 | <0.001 | Middle frontal gyrus, right |
| 77 | 18 40 38 | 3.53 | <0.001 | Superior frontal gyrus, right |
| 286 | -21 -3 22 | 3.53 | <0.001 | Caudate, left |
| 42 | -27 51 -3 | 3.28 | <0.001 | Superior frontal gyrus, left |
| **Perimenopause > Premenopause** | | | | |
| 134 | -28 24 32 | 3.51 | <0.001 | Superior frontal gyrus, left |

*Voxel-level *P* < 0.001, uncorrected. Analyses are adjusted by age, plasma estradiol and sex hormone binding globulin levels.

## Supplementary Table 3. Age effects on regional brain ER density

|  | Overall | P | Premenopause | P | Perimenopause | P | Postmenopause | P |
| --- | --- | --- | --- | --- | --- | --- | --- | --- |
| Amygdala | 0.443 | **0.020** | 0.514 | 0.223 | 0.310 | 0.721 | 0.117 | 0.944 |
| Caudate | 0.343 | 0.108 | 0.048 | 0.977 | -0.178 | 0.937 | 0.173 | 0.937 |
| Hippocampus | 0.534 | **0.003** | 0.513 | 0.223 | 0.271 | 0.721 | 0.388 | 0.480 |
| Hypothalamus | -0.033 | 0.977 | 0.059 | 0.977 | -0.498 | 0.236 | 0.295 | 0.721 |
| Inferior frontal | 0.444 | **0.020** | 0.283 | 0.721 | 0.173 | 0.937 | 0.029 | 0.988 |
| Middle frontal | 0.254 | 0.275 | 0.085 | 0.977 | -0.074 | 0.977 | -0.141 | 0.944 |
| Pituitary | 0.302 | 0.208 | -0.272 | 0.721 | -0.063 | 0.977 | -0.026 | 0.988 |
| Posterior cingulate | 0.400 | **0.035** | 0.196 | 0.937 | -0.108 | 0.966 | 0.121 | 0.944 |
| Thalamus | 0.409 | **0.035** | -0.169 | 0.937 | 0.133 | 0.944 | 0.267 | 0.721 |

Partial correlation coefficients adjusting by plasma estradiol and sex hormone binding globulin levels. Significant P values are in bold.

## Supplementary Table 4. Age by menopause status effects on regional brain ER density

| **Region** | **Interaction** | **Estimate** | **P value** |
| --- | --- | --- | --- |
| Amygdala | Peri vs Pre | -0.0062229 | 0.3263112 |
|  | Post vs Pre | -0.0072135 | 0.2657155 |
|  | Post vs Peri | 0.0009905 | 0.8452813 |
| Caudate | Peri vs Pre | -0.0023156 | 0.7591332 |
|  | Post vs Pre | -0.0010688 | 0.8896782 |
|  | Post vs Peri | 0.0012468 | 0.8374388 |
| Hippocampus | Peri vs Pre | -0.0081322 | 0.1584208 |
|  | Post vs Pre | -0.0070454 | 0.2398076 |
|  | Post vs Peri | -0.0010867 | 0.8213763 |
| Hypothalamus | Peri vs Pre | -0.0177680 | 0.0777467 |
|  | Post vs Pre | -0.0078559 | 0.4381974 |
|  | Post vs Peri | -0.0099121 | 0.2172812 |
| Inferior frontal | Peri vs Pre | -0.0016524 | 0.7667536 |
|  | Post vs Pre | -0.0055228 | 0.3332692 |
|  | Post vs Peri | 0.0038704 | 0.3895390 |
| Middle frontal | Peri vs Pre | -0.0012635 | 0.8506366 |
|  | Post vs Pre | -0.0046839 | 0.4950889 |
|  | Post vs Peri | 0.0034204 | 0.5273955 |
| Pituitary | Peri vs Pre | 0.0486116 | 0.4289055 |
|  | Post vs Pre | 0.0604787 | 0.3357658 |
|  | Post vs Peri | -0.0118671 | 0.8097868 |
| Posterior cingulate | Peri vs Pre | -0.0071596 | 0.3573535 |
|  | Post vs Pre | -0.0013877 | 0.8606089 |
|  | Post vs Peri | -0.0057719 | 0.3566122 |
| Thalamus | Peri vs Pre | 0.0036274 | 0.6494527 |
|  | Post vs Pre | 0.0029669 | 0.7155723 |
|  | Post vs Peri | 0.0006605 | 0.9180182 |

Abbreviations: Peri, perimenopausal group; Post, postmenopausal group; Pre, premenopausal group. Estimates from multivariable regression models testing for effects of age, menopause status, and their interactions on regional estrogen receptor density, adjusting by confounders.
